# Vulnerable Parts of the Human Anatomy

**Published:** 1898-12

**Authors:** 


### VULNERABLE PARTS OF THE HUMAN ANATOMY.

The murderers have discovered some astonishingly vulnerable parts of the human anatomy of late. From a paper this morning we learn that a Georgia colonel was "shot in the ticket office;" the other day a man was fatally shot "through his door," and not long ago another received a fatal wound "in his window."—*New York Commercial Advertiser.*

He kissed her passionately upon her reappearance.—*Jefferson Souvenir.*

She whipped him upon his return.—*Hawkeye.*

He kissed her back.—*Constitution.*

She seated herself upon his entering.—*Albia Democrat.*

We thought she sat down upon her being asked.—*Saturday Gossip.*

She fainted upon his departure.—*Lynn Union.*

He kicked the tramp upon his sitting down.—*American Pharmacist.*

We feel compelled to refer again to the poor woman who was shot in the oil regions some time ago.—*The Medical World.*

And why not drop a tear for the man who was fatally stabbed in the rotunda, and for him who was kicked on the highway? For all the above we are indebted to the *Medical Age*, but it fails to mention the fact of the woman being accidently shot in the water-works, or the man injured upon the long bridge.—*Colorado Medical Journal.*

